# Absolute dose determination in high-energy electron beams: Comparison of IAEA dosimetry protocols

**DOI:** 10.4103/0971-6203.42754

**Published:** 2008

**Authors:** S. Sathiyan, M. Ravikumar

**Affiliations:** Department of Radiation Physics, Kidwai Memorial Institute of Oncology, Bangalore, India

**Keywords:** Absorbed dose, chamber, cross-calibration, protocol

## Abstract

In this study, absorbed doses were measured and compared for high-energy electrons (6, 9, 12, 16, and 20 MeV) using International Atomic Energy Agency (IAEA), Technical Reports Series No. 277 (TRS), TRS 381, and TRS 398 dosimetry protocols. Absolute dose measurements were carried out using FC65-G Farmer chamber and Nordic Association of Clinical Physicists (NACP) parallel plate chamber with DOSE1 electrometer in WP1-D water phantom for reference field size of 15 × 15 cm^2^ at 100 cm source-to-surface distance. The results show that the difference between TRS 398 and TRS 381 was about 0.24% to 1.3% depending upon the energy, and the maximum difference between TRS 398 and TRS 277 was 1.5%. The use of cylindrical chamber in electron beam gives the maximum dose difference between the TRS 398 and TRS 277 in the order of 1.4% for energies above 10 MeV (R_50_ > 4 g/cm^2^). It was observed that the accuracy of dose estimation was better with the protocols based on the water calibration procedures, as no conversion quantities are involved for conversion of dose from air to water. The cross-calibration procedure of parallel plate chamber with high-energy electron beams is recommended as it avoids p_wall_ correction factor entering into the determination of k_Q,Qo_.

## Introduction

Advances in radiation dosimetry continue to improve the accuracy of calibrating photon and electron beams of radiation therapy. With the improved anatomical information obtained from sophisticated diagnostic imaging procedures, the data required to achieve better accuracy in patient treatment depends upon the measured dose. The success of radiotherapy depends on the absorbed dose delivered to the tumor, and it should not vary with respect to prescribed dose by more than ±5%.[1] This requires that the overall uncertainties in radiation dosimetry be minimized, which can be achieved by implementation of uniform measurement procedures in calibration laboratories and user beams. Since it is possible to delineate the target and other critical structures using sophisticated diagnostic imaging procedures, there is a need to evaluate the absorbed dose accurately to maximize the target dose and minimize the normal tissue dose.

The IAEA in collaboration with other international organizations (WHO, PAHO, and ESTRO) has developed various protocols for high-energy electron beams, like absorbed dose determination in photon and electron beams,[2] the use of parallel plate chambers in high-energy electron and photon beams,[3] and absorbed dose determination in external beam radiotherapy.[4] American Association of Physicists in Medicine has also developed various task groups for high-energy electron beams, like AAPM TG-21, AAPM TG-39, and AAPM TG-51.[5–7] According to TRS 277 and TG 21 protocols, the absorbed dose at a specified depth can be calculated using air-kerma calibration factor obtained from the cobalt therapy beam for all electron beams used clinically. IAEA TRS 381 protocol recommends the use of parallel plate chamber to determine absorbed dose in high-energy photon and electron beams using air chamber calibration factor (N_D,air_^pp^). Recent protocols TG 51 and TRS 398 suggest the measurement of absorbed dose in phantom at a reference point, using absorbed dose to water calibration factor (N_D,w_). At present, Secondary Standard Dosimetry Laboratories (SSDL) does not provide calibration factors for all user beam qualities. They provide calibration factor only for ^60^Co beam. Quality specific conversion factor is to be used to determine absorbed dose to water for the interested beam qualities based on the SSDL reference calibration factor.

Ever since the ‘absorbed dose to water’ concept was introduced, a frequent question has been raised about the difference between water-and air-kerma-based protocols. Several authors have compared different protocols to study the various aspects influencing the accuracy of delivered dose.[8–12] The IAEA-based recommendations of TRS 398 differ significantly from TRS 277 and TRS 381. These significant differences are contributions from difference in the calibration factor and stopping power ratios. The IAEA[11] reported on experimental comparison of high-energy electron beam dosimetry using TRS 277, TRS 381, TRS 398, TG 51, and DIN 6800-2 protocols. The test results are reported and compared in the document using the above protocols with various types of chambers. It has been found that the maximum differences in absorbed dose determination between TRS 398 and the previous Codes of Practice TRS 277 (2^nd^ ed.) and TRS 381 are of the order of 1% to 2%, depending on the energy and the detector system used. In this study, TRS 277, TRS 381, and TRS 398 protocols were compared to evaluate the absolute dose measurements in high-energy electron beams using parallel plate and cylindrical ion chambers. The parallel plate chamber was cross-calibrated against the cylindrical chamber, and the dose measurements carried out with the same were compared.

## Materials and Methods

**Figure 1:**
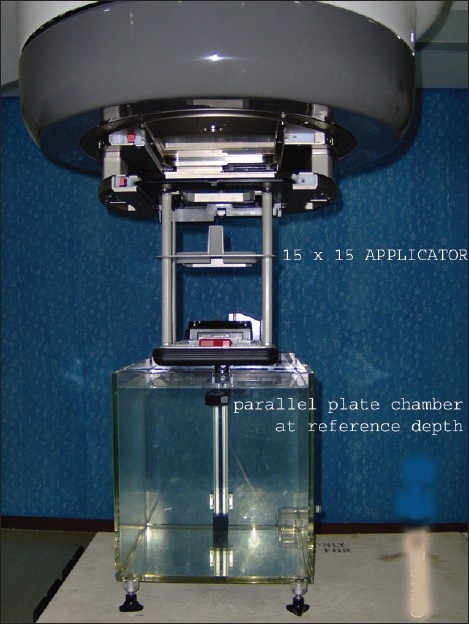
Experimental setup

High-energy electron beams of 6, 9, 12, 16, and 20 MeV from Clinac-DHX (Varian Medical Systems, Palo Alto, CA, USA) dual-energy photon linear accelerator were used in this study. Absolute dose measurements were carried out using DOSE1 electrometer (Wellhofer, Scanditronix) with 0.65 cm^3^ (FC65-G) Farmer-type ion chamber and NACP-02 parallel plate chamber of volume 0.16 cm^3^. The front window thickness of parallel plate chamber was 0.5 mm of graphite (0.6 mm with Mylar foil for water protection). No leakage was observed in the chamber and/or the electrometer during measurements. The measurements were carried out in 30×40×30 cm^3^, WP1-D manual water phantom (Scanditronix) according to TG 51 and IAEA TRS 398 dosimetry protocols. The measurement depth can be manually adjusted with 0.1 mm steps, and the depth of measurement was read out on the incremental encoder with integrated display. All measurements were carried out at reference depth using the reference standard applicator of size of 15×15 cm^2^ provided by the manufacturer. The measurement setup used in our study is shown in Figure 1. All measurements were done by strictly adhering to the conditions stipulated in the protocols. Three measurements were made to minimize the statistical uncertainty in dose measurement. The ion recombination and polarity effects have been measured and corrected for each value of electron energy. For electron dosimetry, all the protocols recommend a cross-calibration for parallel plate chamber against calibrated cylindrical chamber. The rationale for this is the large uncertainty in the wall perturbation factor p_wall_ at ^60^Co energy for different makes of parallel plate chambers. Hence cross-calibration procedure was also carried out in this study.

### Energy parameters

In TRS 277, the range-energy relationship is strictly valid for depth absorbed dose distributions. The measurement of R_p_ and R_50_ are necessary to determine the most probable energy at the surface (Ē_P,0_) and the mean energy at the phantom surface (E_0_). It is given by

[1] $${Ē}_{P,0}=C_{1}+C_{2}R_{p}+C_{3}{R_{p}}^{2}$$

where C_1_ = 0.22 MeV, C_2_ = 1.98 MeV cm^−1^, C_3_ = 0.0025 MeVcm^−2^.

[2] $${Ē}_{0}=C_{4}R_{50}$$

where C_4_ = 2.33 MeV·cm^−^1 Mean energy as a function of depth is given by

[3] $${Ē}_{z}=E_{0}(1-z/R_{p})$$

where R_p_ is the practical range, which is defined as the depth where the tangent to the descendent part of the curve intersects the prolongation of the bremsstrahlung tail, and R_50_ is the depth where the absorbed dose is 50% of the maximum dose.

In TRS 381, the equation for the mean energy at the phantom surface (Ē_0_) is valid for large field sizes of electron energies 5 to 30 MeV, and for R_50_ determined from depth-dose distributions measured in water. Ē_0_ can be determined from ionization curve or depth-dose curve measured at 100 cm SSD with an ionization chamber or a solid state detector using the relationship

[4] $${Ē}_{0}[\mathrm{MeV}]=0.818+1.935R_{50}^{J}+0.040{\left(R_{50}^{J}\right)}^{2}$$

for r^J^_50_ determined from a depth-ionization curve;

[5] $${Ē}_{0}[\mathrm{MeV}]=0.656+2.059R_{50}^{D}+0.022{\left(R_{50}^{D}\right)}^{2}$$

for R^D^_50_ determined from a depth-dose curve.

In TRS 398, the mean energy at the phantom surface is given by Ē_0_ = 2.33 R_50_ MeV and R_50_ expressed in g/cm^2^.

### 2. Overview of formalism of various IAEA codes of practice for electron beams

A summary of the formalism in the various IAEA codes of practice protocols is presented in order to establish a parallelism among them. The original notations used by the various codes of practice (CoPs) and protocols for various interaction coefficients, influential quantities, and perturbation correction factors will be retained in the discussion of the present section. However, in the subsequent sections, the notations given in the TRS 398,[4] TRS 277,[2] and TRS 381[3] will mostly be used.

### 2.1. IAEA TRS 277

Determination of absorbed dose to water at reference depth in a phantom is a two-step process. In the first step, a chamber factor in terms of the absorbed dose to the cavity air, N_D_, is derived:

[6] $$N_{D}=N_{K}(1-g)k_{\mathrm{att}}k_{m}$$

where k_m_ is the factor to take into account the non-air equivalence of the ionization chamber, ionization chamber wall, and buildup cap material. In the second step, the absorbed dose to water, D_w,Q_, at a point in a phantom where the effective point of measurement of the chamber is positioned, is obtained from the dose to the cavity air using the Bragg-Gray principle,

[7] $$D_{w}(p_{\mathrm{eff}})=M_{u}.p_{\mathrm{TP}}.N_{D}.k_{h}.k_{s}.{\left(s_{w,\mathrm{air}}\right)}_{u}.p_{u}$$

where M_u_ is the meter reading, P_TP_ is the factor to allow for effects of nonreference temperature and pressure, and N_D_ is the absorbed dose to air chamber factor. The humidity correction is represented by k_h,_ k_s_ is the ion recombination correction, s_w,air_ is the stopping power ratio for the electron energy, and p_u_ is the perturbation correction factor. The effective point of measurement, p_eff_, is 0.5r (i.e., z_peff_ − z_p_ = 0.5r) upstream from the center of the chamber for cylindrical chambers, and for plane parallel plate chamber, it is at the front surface of the air cavity.

### 2.2. IAEA TRS 381

There are two approaches to determine absorbed dose to water in high-energy electron beam quality Q, depending on whether chamber has N_D,air_ or N_D,w_ calibration factor.

#### 2.2.1. Dosimetry with N_D,air_ calibration factor for parallel plate chamber

Absorbed dose to water D_w,Q_ for the beam quality Q, at the effective point of measurement P_eff_ positioned at reference depth, is given by

[8] $$D_{w,Q}(p_{\mathrm{eff}})=M_{Q}.N_{D,\mathrm{air}}.{\left(s_{w,\mathrm{air}}\right)}_{Q}.{\left(p_{\mathrm{cav}}p_{\mathrm{wall}}\right)}_{Q}$$

where M_Q_ = M_l._ P_TP_· P_s_·

M_l_ is the meter reading, P_TP_ is the factor to allow for effects of nonreference temperature and pressure, and N_D,air_ is the absorbed dose to air chamber factor. The ion recombination correction factor is P_s_, the stopping power ratio of water to air is (s_w,air_)_Q_. p_Q_ is the overall perturbation factor (p_cav_ p_wall_), perturbation due to air cavity is p_cav_, and p_wall_ is the effect due to non-air equivalence of chamber wall material.

#### 2.2.2. Dosimetry with N_D,w_ calibration factor for parallel plate chamber

When the parallel plate chamber has absorbed dose to water calibration factor, absorbed dose to water at the effective point of measurement is

[9] $$D_{w,Q}(p_{\mathrm{eff}})=M_{Q}.N_{D,w,\mathrm{Qo}}.k_{Q}$$

where N_D,w,Qo_ is the absorbed dose to water calibration factor at reference beam quality, k_Q_ is the beam quality conversion factor.

The reference depth in water phantom for absorbed dose determination in electron beams is R^D^_100_ for energies less than 5 MeV, R^D^_100_ or 1 cm for energies ranging from 5 to less than10 MeV, R^D^_100_ or 2 cm for energies ranging from 10 to less than 20 MeV, and R^D^_100_ or 3 cm for energies ranging from 20 to less than 50 MeV. As suggested by the protocol, larger depth was selected for the measurement depending on the energy.

### 2.3. IAEA TRS 398

#### 2.3.1. Dosimetry with N_D,w_ calibration factor for cylindrical and parallel plate chambers (calibration in Co-60 beam)

The absorbed dose to water at the reference depth z_ref_ in water, in an electron beam quality Q is

[10] $$D_{w,Q}=M_{Q}.N_{D,w,\mathrm{Qo}}.k_{Q,\mathrm{Qo}}$$

where M_Q_ = M_l_. h_pl_. k_TP_. k_elec_. k_pol_. k_s_

M_l_ is the uncorrected dosimeter reading, h_pl_ is the fluence scaling factor (for water, h_pl_ =1), k_TP_ is the pressure temperature correction factor. The electrometer calibration factor is k_elec_, k_pol_ is the polarity correction factor, and k_s_ is the recombination correction factor. The polarity correction factor k_pol_ is given by

[11] $$k_{\mathrm{pol}}=\frac{\left|M+|+|M-\right|}{2M}$$

where M _+_ is the meter reading for polarizing voltage +V, and M _−_ is the meter reading for polarizing voltage −V. The recombination correction factor k_s_ is given by

[12] $$k_{s}=a_{0}+a_{1}\left(M_{1}/M_{2}\right)+a_{2}{\left(M_{1}/M_{2}\right)}^{2}$$

where M_1_ and M_2_ are the meter readings obtained at two different bias voltages V_1_ and V_2_ for the same irradiation condition. The constants a_0_, a_1_, and a_2_ are the voltage ratio dependents, which can be obtained from the protocol.

N_D,w,Qo_ is absorbed dose to water calibration factor at the reference beam quality Q_o_, and k_Q,Qo_ is the chamber-specific factor which corrects for difference between the reference beam quality Q_0_ and the actual beam quality Q. The reference depth (z_ref_) is 0.6 R_50_ − 0.1 g/cm^2^. The position of the reference point of the chamber for parallel plate is at z_ref_; and for cylindrical chamber, at 0.5 r_cyl_ deeper than z_ref_, where z_ref_ is the center of the chamber.

#### 2.3.2. Cross-calibration of parallel plate chamber in electron beam

The parallel plate chamber was cross-calibrated against a reference cylindrical chamber with an electron beam of energy 20 MeV having an R_p_ of 8.3 g/cm^2^. The reference chamber and the chamber to be calibrated were compared by alternately positioning each other at the reference depth z_ref_ in water. The calibration factor in terms of absorbed dose to water for the chamber under calibration at the cross-calibration quality Q_cross_ is

$${N^{x}}_{D,w,\mathrm{Qcross}}=\frac{{M^{\mathrm{ref}}}_{\mathrm{Qcross}}}{{M^{x}}_{\mathrm{Qcross}}}{N^{\mathrm{ref}}}_{D,w,\mathrm{Qo}}.{k^{\mathrm{ref}}}_{\mathrm{Qcross},\mathrm{Qo}}$$

where M^ref^_Qcross_ is the dosimeter reading for reference chamber, M^x^_Qcross_ is the dosimeter reading for chamber to be calibrated, N^ref^_D,w,Qo_ is the absorbed dose to water calibration factor for reference chamber, and k^ref^_Qcross,Qo_ is the beam quality conversion factor for reference chamber.

The absorbed dose to water for user beam quality can be determined from the above calibration factor

[14] $$D_{w,Q}={M^{x}}_{Q.}{N^{x}}_{D,w,\mathrm{Qcross}.}{K^{x}}_{Q,\mathrm{Qcross}}$$

where

[15] $${K^{x}}_{Q,\mathrm{Qcross}}=\frac{{K^{x}}_{Q,\mathrm{Qint}}}{{K^{x}}_{\mathrm{Qcross},\mathrm{Qint}}}$$

M^x^_Q_ is the meter reading corrected for influential quantities.

Such a calibration generally results in determination of absorbed dose to water using parallel plate chamber that is more reliable than that achieved by use of parallel plate chamber directly calibrated in ^60^Co, mainly because of problems associated with the p_wall_ correction for plane-parallel chambers in ^60^Co, entering into the determination of k_Q,Qo_. Table 1 shows the measurement depth used in various protocols. The calibration factors and the associated correction factors for the chambers used in this study are shown in Table 2 and 3. Table 4 shows the summary of the calibration of chambers for dosimetry in high-energy electron beams according to the IAEA Technical Reports.

**Table 1:** Measurement depth z_ref_ used in various protocols

*Energy*	*Measurement depth z_ref_ (g / cm^2^)*		
	
	*TRS 277*	*TRS 381*	*TRS 398*
6 MeV	1.33	1.33	1.33
9 MeV	2.11	2.11	2.05
12 MeV	2.85	2.85	2.89
16 MeV	3.23	3.23	3.85
20 MeV	2.31	2.31	4.88

**Table 2:** Calibration factors for the chambers used in this study

*Chambers*	*N_D,air_(^60^Co) factor*	*N_D,w_ (^60^Co) factor*	*N_D,w_ (cross-calibration) factor*
NACP Plane parallel chamber	1.4422 × 10^8^ Gy / C	1.634 × 10^8^ Gy / C	1.4591 × 10^8^ Gy / C
FC65-G Farmer chamber	4.2409 × 10^7^ Gy / C	4.805 × 10^7^ Gy / C	——

**Table 3:** Various correction factors as a function of energy and chamber

Energy	NACP Plane parallel chamber				FC65-G Farmer chamber			
		
	k_Q,Q0_	Ion recombination (k_s_)	Polarity effect (k_pol_)	Stopping power ratio (s_w,air_)	k_Q,Q0_	Ion recombination (k_s_)	Polarity effect (k_pol_)	Stopping power ratio (s_w,air_)
6 MeV	0.926	1.008	0.998	1.079	—	—	—	—
9 MeV	0.911	1.009	0.999	1.050	—	—	—	–
12 MeV	0.899	1.009	1.000	1.037	0.916	1.013	1.001	1.037
16 MeV	0.888	1.001	1.003	1.010	0.911	1.012	1.000	1.010
20 MeV	0.879	1.010	1.000	0.977	0.906	1.013	1.000	0.977

**Table 4:** Calibration of chambers for dosimetry with high-energy electron beams according to the IAEA technical reports

Report	Calibration	Phantom material	Beam	Remarks
TRS 277 (Update 1997)	Absorbed dose to air	Air	Co-60	Calibration laboratory
TRS 277 (Update 1997)	Absorbed dose to water	Water	Co-60	Calibration laboratory
TRS 381 (1997)	Absorbed dose to air	Air	Co-60	Only for plane-parallel chambers, Calibration laboratory
TRS 381 (1997)	Absorbed dose to air	Plastic	Co-60	Only for plane-parallel chambers, Calibration laboratory or User
TRS 381 (1997)	Absorbed dose to air	Water	Electron beam	Only for plane-parallel chambers, Calibration laboratory or User
TRS 381 (1997)	Absorbed dose to water	Water	Co-60	Only for plane-parallel chambers, Calibration laboratory or User
TRS 398 (2000)	Absorbed dose to water	Water	Co-60	Calibration laboratory
TRS 398 (2000)	Absorbed dose to water	Water	Electron beam	Only for plane-parallel chambers, “cross-calibration” user

## Results

**Figure 2:**
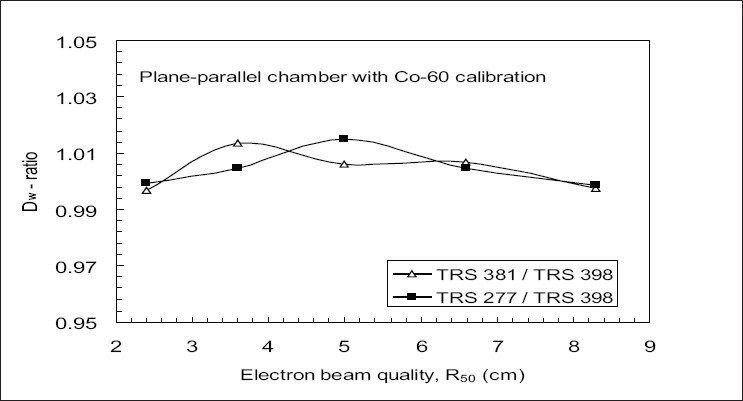
Experimental comparison of dose ratios of IAEA TRS protocols in electron beams, at the depth of maximum dose for plane-parallel ion chamber having N_D,w_ calibrations in ^60^Co

Figure 2 shows the experimental comparison of dose ratios TRS 277 / TRS 398 and TRS 381 / TRS 398 in electron beams at the depth of dose maximum for NACP parallel plate chamber having N_D,w_ calibration factor in ^60^Co. The maximum difference between TRS 381 and TRS 398 was 1.3%, and the maximum difference between TRS 277 and TRS 398 was found to be 1.5%.

**Figure 3:**
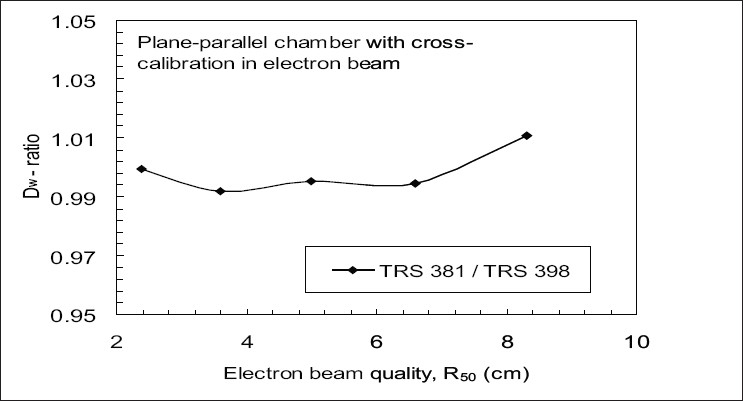
Experimental comparison of dose ratios TRS 381 / TRS 398 in electron beams, at the depth of maximum dose for plane-parallel ion chamber cross calibrated in high energy electron against Farmer type ion chamber

Figure 3 shows the experimental comparison of the dose ratio TRS 381 / TRS 398 for the electron beams at the depth of dose maximum for NACP parallel plate chamber. These results have been obtained by cross-calibration of parallel plate chamber in the high-energy electron beams against the Farmer-type chamber having N_D,W_ calibration factor in ^60^Co beam. The maximum deviation in the measured absorbed dose with the two protocols was 1.1%.

**Figure 4:**
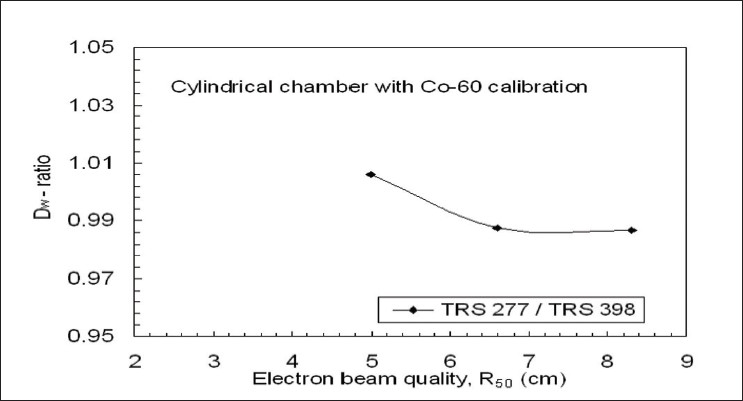
Experimental comparison of dose ratios TRS 277 / TRS 398 in electron beams, at the depth of maximum dose for cylindrical ion chamber having N_D,w_ calibrations in ^60^Co

Figure 4 shows the plot of the dose ratio between TRS 277 and TRS 398 (TRS 277 / TRS 398) as a function of R_50_ for the electron beam dosimetry, using Farmer-type ion chamber with N_D,w_ calibrated at ^60^Co. The maximum dose difference was 1.4%.

## Discussion and Conclusion

The result shows that the absorbed dose variation between TRS 381 and TRS 398 protocols was in the range of 0.24% to 1.3%, depending upon the electron energy. The maximum dose difference between TRS 277 and TRS 398 protocols was 1.5%. The IAEA-TECDOC^11^ shows that maximum difference between TRS 398 and TRS 381 is of the order of 1% for NACP and Roos PTW commercial chambers; for the Roos PTB prototype, the maximum discrepancy is up to 1.5% at the lowest and highest energies. It is also reported that the dose ratio TRS 398 and TRS 277 is up to 2%. From this study, it was observed that the maximum deviation in the measured absorbed dose with TRS 398 and TRS 381 was 1.1% for NACP parallel plate having N_D,w_ cross-calibration factor. The IAEA-TECDOC report has indicated the maximum deviation of 1.3% at higher energies, which is in agreement with our results.

The IAEA-TECDOC^11^ has reported that the maximum differences in absorbed dose determination between TRS 398 and the previous Codes of Practice TRS 277 (2^nd^ ed.) and TRS 381 are of the order of 1% to 2%. The report recommends that the users are advised to check carefully their experimental conditions and relevant calibration coefficients if the ratios of absorbed doses, D_w_ (TRS 398) / D_w_ (other CoPs), measured by them fall outside the range recommended by this report. The dose ratios of TRS 398 in comparison with other codes of practice (TRS 381 and TRS 277) were in good agreement with IAEA-TECDOC-1455. The accuracy of dose estimation would be more with the protocols based on the water calibration procedures, as no conversion quantities are involved for conversion from air to water. The cross-calibration procedure of parallel plate chamber with high-energy electron beams is recommended as it avoids P_wall_ correction factor entering into the determination of k_Q,Qo_.
